# Variant cell types and host glycoproteins influencing HIV-1 infection

**DOI:** 10.1186/1742-4690-8-S2-O28

**Published:** 2011-10-03

**Authors:** William A Paxton

**Affiliations:** 1 University of Amsterdam, the Netherlands

## 

A number of variant cell types are associated with HIV-1 transmission and disease progression, including dendritic cells (DCs), macrophages and CD4 lymphocytes. The cell types and their phenotypes associated with infection are still poorly defined and will greatly influence HIV-1 transmission as well as disease progression. The types of immune responses mounted against infectious pathogens will also influence the pool of cells to be infected and thereby influence disease course. We have described CD4 lymphocyte cellular phenotypes induced within the same HIV-1 infected individuals against variant pathogens (namely CMV and TB antigens) and shown that these responses are highly variable. We further demonstrate that their distinctive cytokine and chemokine expression patterns correlate to their HIV-1 infectivity profile both in vitro and in vivo. We have additionally addressed whether viruses produced from variant cellular populations have similar or distinctive phenotypes. Here we show that viruses produced from macrophages opposed to Th1 or Th2 CD4 lymphocytes vary for their interaction with DC-SIGN expressed on DCs and have variant neutralization profiles for 2G12. Furthermore, we have identified a number of host glycopoteins (BSSL and MUC6) that can inhibit HIV-1 from interacting with DCs through binding DC-SIGN. We show that genetic polymorphisms within the BSSL gene can associate with strength of binding to DC-SIGN and that these variants can also be associated with markers of disease progression as well as time to coreceptor switch. In conclusion, the interactions between the adaptive and innate immune response can influence HIV-1 transmission and modulate disease course.

